# Donor Blood Procurement, Safety, and Clinical Utilization: A Study of Blood Transfusion Services in a Tertiary Care Hospital in Nigeria

**DOI:** 10.1155/2022/2622291

**Published:** 2022-03-17

**Authors:** Oluomachi Charity Nnachi, Charles Uzor, Chukwuma David Umeokonkwo, Emeka Ogah Onwe, Augustine Ejike Okoye, Richard Lawrence Ewah, Favour Ogonna Nwani

**Affiliations:** ^1^Department of Haematology, Alex Ekwueme Federal University Teaching Hospital, Abakaliki, Ebonyi State, Nigeria; ^2^Department of Community Medicine, Alex Ekwueme Federal University Teaching Hospital, Abakaliki, Ebonyi State, Nigeria; ^3^Department of Paediatrics, Alex Ekwueme Federal University Teaching Hospital, Abakaliki, Ebonyi State, Nigeria; ^4^Department of Anaesthesia, Alex Ekwueme Federal University Teaching Hospital, Abakaliki, Ebonyi State, Nigeria

## Abstract

**Background:**

Donated blood is an essential component of the management of many diseases, and hospital-based blood banks in Nigeria are saddled with the responsibility of provision of safe blood and coordination of its appropriate utilization for patient care.

**Objective:**

This study reviewed the extent to which the hospital blood transfusion service ensures adequate safe blood supply and utilization. *Materials/Methods*. This was a retrospective study of 2 years record of the blood bank service of Alex Ekwueme Federal University Teaching. Methods of donor blood procurement, transfusion transmissible infection status, the pattern of blood, and blood component usage across the hospital's clinical departments were evaluated. Statistical analysis was conducted using IBM SPSS, and data were presented as percentages. Fisher's tests were used to test significance, and *p* value <0.05 is significant.

**Results:**

The highest proportion of donors was male family replacement donors aged 26–35 years (3634 (39.68%)) while total voluntary donors were 315 (2.65%). Hepatitis B had the highest seroprevalence 267 (2.22%) among blood-borne diseases screened. National Blood Transfusion Service (NBTS) supplied only 3 (0.03%) of total blood units used. The accident and emergency department had the highest proportion of persons who utilized whole blood; 4568 (99.96%).

**Conclusion:**

The hospital blood bank relies heavily on family replacement donors with little or no assistance from the National Blood Transfusion Service. Family replacement donors have the highest risk of TTIs, and hepatitis B infection has the highest prevalence. The high cost of blood component therapy increases the need for whole blood.

## 1. Introduction

Blood transfusion is the most commonly performed procedure in healthcare facilities [1–3]. Demand for blood is high; blood donation rates are very low, especially in low- and middle-income countries in Africa. Transfusion medicine has evolved from a laboratory service to clinical care with a focus on blood safety and appropriate clinical use of blood. The imperative goal is to improve clinical outcomes and patient safety. However, despite compelling evidence and ongoing WHO policy drive, Blood transfusion safety in Nigeria has been challenged by a shortage of voluntary donors, poor infrastructure, high cost of blood components, and high prevalence of transfusion transmissible infections (TTIs) [4].

Transfused blood is a known risk for transmission of infectious diseases including hepatitis B, C, and HIV. The prevalence of hepatitis B infection among blood donors in Nigeria has been variable. A study reported the prevalence of HBV, HCV, syphilis, and HIV to be 4.1%, 3.6%, 3.1%, and 4.2% among donors while another study reported rates as high as 17% for hepatitis B seropositivity and as low as 0.45% for HIV [5–9].

Donorship rates for voluntarily donated blood are very low in Nigeria. The National Blood Transfusion Service, which is the agency responsible for safe blood supply in Nigeria is unable to meet more than 75% of the blood needs of the populace [10].

The effectiveness and safety of hospital blood transfusion services in our country with a low human development index are critical to healthcare delivery. This study seeks to define the extent to which the hospital blood transfusion service ensures adequate safe blood supply and utilization, at a tertiary hospital in southeast Nigeria.

### 1.1. Subjects, Materials, and Methods

This was a retrospective study conducted at the Department of Haematology at the Alex Ekwueme Federal University Teaching Hospital, Abakaliki, Ebonyi State (AEFUTHA). Ethical approval was obtained from the Ethical and Research Committee of AEFUTHA (REC protocol no. 19/04/2021–10/06/2021). Records from the blood transfusion unit and hospital ward blood transfusion register from January 1, 2019, to December 31, 2020, were collated and analyzed to identify the methods of donor blood donation, demographic data (age and sex) of blood donors during the specified study period, the various categories of blood donors, the prevalence of TTI markers, and pattern of blood and blood component usage across the hospital's clinical departments.

No autologous donation was done during the study period. The sources of blood procurement are paid blood donors, voluntary donors, and family replacement donors. A paid blood donor offers a unit of blood for a pecuniary benefit by a contracted hospital vendor. A replacement blood donor is a family member or relative of a patient, donating a unit of blood to be used for a specific patient, while a voluntary blood donor is a member of the society who donates his or her blood without any inducement for use by a recipient not known to him or her. Commercially donated blood is supplied to the hospital blood bank by contracted vendors while blood from voluntary donors and replacement donors are collected in the hospital donor clinic after packed cell volume, blood group, and other eligibility criteria are determined. Thereafter, sera samples are tested for hepatitis B surface antigen (HBsAg), antibodies to hepatitis C virus (HCV), human immunodeficiency virus (HIV) 1/2, and *Treponema pallidum* using commercially available immunochromatic based rapid kits. A single red line at position *C* (control) on the strip indicates a valid control. A test is positive if two transverse bands (*T* = test and *C* = control) are seen and negative when only the one band at control (*C*) is seen. Those who test positive are disqualified. Blood (450 ml) is usually collected into a bag containing 63 ml citrate phosphate dextrose with adenine (CPDA) using the standard guidelines for routine phlebotomy and stored at 2–8°C as whole blood or separated into components (fresh plasma, red cell concentrate, or platelet-rich plasma) using the cold centrifuge machine. Donors for platelet concentrate have their predonation platelet count measured (predonation platelet count of at least 200,00/*μ*l required) and blood group determined since platelet concentrate transfusion is blood group-specific and prepared only on demand due to cost. The Haemonetics MCS apheresis machine is used for platelet concentrate harvest from the donors. They are stored in the platelet agitator. Whole blood supplied by vendors (paid donors) are also tested for, hepatitis B, C, *Treponema pallidium*, and HIV 1 and 2 after supply and if found to be negative for all fours tests, they are then stored in the blood bank refrigerator for use.

Statistical analysis was conducted using IBM SPSS, and data were presented as percentages. Fisher's tests used to test significance, and *p* value <0.05 is significant.

## 2. Results

Table 1 shows the age and sex distribution of persons who donated blood during the study period. The majority of blood donors were males (12,268 (97.11%)) amongst which those aged 26 to 35 years were the highest proportion (4,729 (38.47%)).

Table 2 shows the age and sex distribution of study donors across the categories of blood donation. The highest proportion of donors were male family replacement donors (3,634 (39.68%)) and female replacement donors (143 (43.86%)) aged 26 to 35 years. Voluntary donors contributed 315 (2.65%) of all the categories of blood donors.

Family replacement donors had the highest number of rejection/deferral 358 (59.6%) while low haemoglobin was the commonest reason for rejection/deferral 444 (73.8%).

Table 3 shows the distribution of blood groups across persons who donated blood during the study period. The majority of donors were O Rh-positive (8,739 (73.1%)) followed by A Rh-positive (1,411 (11.8%)). Only one person had AB Rh-negative blood group.

Table 4 shows the seroprevalence of blood-borne diseases among blood donors screened during the study period. Hepatitis B had the highest seroprevalence (267 (2.22%)) among blood-borne disease screened for *p* value <0.001. HIV had the lowest seroprevalence (101 (0.82%)). Amongst the different categories of donors, the hepatitis B, C, HIV, and syphilis seroprevalence was highest among family replacement donors (2.52%, 2.18%, 0.95%, and 2.22%, respectively) while voluntary donors had the lowest. The relative risk of infection transmission was 2.56 and 1.68 for HIV and hepatitis B infections, respectively.

Table 5 shows source/site of blood donation (hospital, private lab, NBTS, and others).

Hospital screening and blood donation (voluntary, family replacement, and paid) constituted the largest source of blood during the study period (8,655 (74.7%)). Vendor-sourced commercial blood accounted for 2,537 (21.9%). NBTS contributed only 3 (0.03%).

Table 6 shows the pattern of usage of blood and blood products across clinical departments.

A total of 11,581 blood units were used during the period under study. The accident and emergency department had the highest proportion of persons who utilized total whole blood (4,568; 99.96%) while internal medicine used the highest number of platelets and fresh frozen plasma with haematology department (28; 2.29% and 12; 0.98%) and (12; 0.69% and 10; 0.57%), respectively.

## 3. Discussion

Voluntary nonremunerated blood ensures safety, quality, availability, and accessibility of blood transfusion. Our study revealed that family replacement and commercial donors were the major sources of blood during the study period with voluntary unpaid donors contributing only 2.65%. These results are comparable to those obtained in other reports, where family replacement donors and commercial donors made up 99% [11] and 95.3% [12], respectively of donated blood in the hospital blood bank. Family replacement donors are not the best source of blood since these donors are usually under pressure to give blood to save a loved one even when they are not eligible on account of being potential transmitters of TTIs or their health is at risk. A more appalling twist to family replacement blood donation is the fear that many of the so-called family replacement donors may not be true relatives but commercial donors co-opted to act as family replacements [13]. The lack of effective community blood drive programmes could account for the low level of voluntarily donated blood and a functional donor clinic can facilitate the conversion of the huge family replacement donor base to voluntary donors. Misconceptions, lack of information, and a high rate of unemployment have also encouraged commercial blood donation to thrive [14, 15].

Demographic information of blood donors is important for drafting and monitoring recruitment strategies [16]. In Africa, males constitute the majority of blood donors with women constituting less than 30% of the donor population [17]. In keeping with our study also, females accounted for only 3.99% of blood donors in our blood bank and the majority were family replacement donors. Our findings differ starkly from those obtained from studies in developed countries [18, 19] where females accounted for as high as 40% to 55% of the blood donor population despite the prevailing barriers including pregnancy, breastfeeding, menstrual blood loss, low blood haemoglobin concentration, and greater susceptibility to vasovagal reactions. Lack of access to education, cultural beliefs, and economic deprivation are further barriers to female participation in blood donation in our locality. Deferral rates due to anaemia are high in females, especially in developing countries [20]. In our environment, a high incidence of malarial infections in the general population and iron deficiency anaemia is notable [21].

Transfusion transmissible infections pose the greatest threats to blood transfusion safety [22]. Our study revealed that the prevalence of HIV, hepatitis C, and syphilis infections in our family replacement donors was higher than that of donations in low-income countries as reported by the WHO [3]. Even though the prevalence for hepatitis B among the different categories of donors was slightly lower than reported by the WHO, family replacement donors has the highest prevalence rate of 2.52 with a 1.68 risk of being infected. A similar prevalence of hepatitis B infection has been found in another study [23]. The commercial and voluntary donors had lower seroactivity and relative risk of transmission of blood-borne diseases. Family replacement donors may have a higher rate of seroactivity of the TTIs since they constitute the greater proportion of total donors assessed. They are usually under immense pressure to donate blood to save the life of someone known to them in emergent situations and are likely to evade divulging information connected to a risky lifestyle that might lead to denying them eligibility to donate. This is at variance with voluntary donors who tend to be repeat donors for altruistic reasons and so are aware and maintain a lifestyle that makes them always eligible. The low prevalence of TTIs among commercial donors may be due to their understanding that their source of income from blood donation may be cut off if they engage in high-risk behaviors that may make them ineligible to donate blood in the future. This disturbing concept has arisen due to poverty and unemployment and requires urgent steps to curb it including proper education and job creation.

Blood group and rhesus typing are routinely determined in blood donors as they are clinically important in haemolytic transfusion reactions The predominant blood type in our study was type O and the least common was type AB, consistent with other studies in Nigeria [24]. In Eastern and Southern African countries, blood group O dominated the populace, while in Pakistan and some regions in India on the Asian continent, blood group B dominated [25–27]. In addition to being the most common blood group in our population, the high rate of utilization of blood group O is seen where there is an inadequate supply of donor blood, particularly in emergencies when it is commonly used as universal donor units for transfusion to A, B, and AB recipients, This is predicated on the premise that blood group O red cells lack A and B antigens on their cell membrane surfaces, despite the risk of the presence of anti-A and anti-B haemolysins in blood type O donors [28].

Contrary to the report by Enosolease et al. [12], our study revealed that blood utilization was more than supplied by the blood bank. This shortage necessitated patients resorting to blood procurement from peripheral laboratories when the hospital blood bank had none. In concordance to the study by Okocha et al. [11], the Accident and Emergency department had the highest rate of utilization of whole blood followed by the obstetrics and gynaecology department. The high blood use by the accident and emergency department is probably a result of the increased requirement of whole blood for emergent resuscitation due to blood loss. The rising incidence of road traffic accidents in our environment is culpable.

More than 98% of blood used during the study period was whole blood. This contributed to increasing our blood needs. The use of whole blood has continued in resource-limited low- and medium-income countries despite the benefits of component therapy. Component therapy allows several patients to benefit from one unit of donated whole blood thereby maximising its use. Most times fresh whole blood transfusion is a quick and cheap fix for patients requiring platelets or coagulation factors only. This is associated with alloimmunization, overload, and poor treatment outcomes. Transfusion of whole blood rather than the indicated component is a failure of stewardship of the scarce blood resource. From our study, the internal medicine department and the haematology unit had the highest utilization of blood components such as packed red cells and platelets possibly due to the chronicity of diseases that affect distinct blood cell lines and so afford time for the patient to gather funds to procure the required blood component. Also, most haematological premalignant and malignant diseases are associated with dangerously low blood counts that require component therapy to support treatment. The low use of blood component therapy is due to the high cost and lack of infrastructure in our environment [12].

Hospital-based blood donation by family replacement and voluntary donors made up the largest contribution to donor blood used in the hospital during the study period while the National Blood Transfusion Service (NBTS) provided only three units of blood. NBTS is saddled with the responsibility to provide safe, quality blood and blood components in a cost-effective manner and distribution to hospitals throughout the country. Currently, Nigeria needs an average of 1.8 million pints of blood annually to meet the blood transfusion needs of 200 million Nigerians, but only 500,000 units are collected annually by the NBTS, amounting to only 27.7% and leaving a deficit of 73.3%.10 Problems faced by the NBTS include inadequate policy enforcement and funding shortfalls which impact its ability to enlighten more people and increase donor recruitment. The funding challenge hinders activities such as media outreach, advocacy, and public awareness campaigns down to the community level which would have tackled the deeply rooted cultural myths and misconceptions on voluntary blood donation in the country. The gap created by the nonfunctional NBTS is filled by vendor-sourced blood which contributed about one-fifth of the total blood used during the study period reflecting the huge dependence on commercially sourced blood in addition to those who disguise as family replacement donors.

More action is urgently needed from policymakers and stakeholders in strengthening the capacity of the National Blood Transfusion Service to ensure that more units of voluntarily donated blood are supplied to hospitals. Donor education of the populace especially targeting the youths is key to inculcating the right concept and culture of VNR blood donation. Hospital donor clinics to aggressively drive community outreaches, donor drive, and design programmes to encourage family replacement donors to become voluntary donors. Government and hospital managers should gear efforts toward the provision of equipment for component preparation and making it affordable. Also, TTI screening must step up to better and more sensitive screening techniques considering the large volume of blood required by patients.

### 3.1. Study Limitation

This is a retrospective study, and donors/recipients with incomplete data from the blood register were removed from the analysis. The data of most recipients' prehaemoglobin levels and diagnosis were missing and could be included as part of the study.

## 4. Conclusion

Blood donation deficit has been demonstrated in this study, and hospital transfusion services are still largely dependent on commercial donors and family replacement donors as the predominant source of blood and blood products utilized by the hospital. The utilization of whole blood was increased due to the nonaffordability of blood components. Moving forward, the National blood transfusion service must become proactive in using results of donor behaviour studies and implementation of actionable policies to improve safe blood supplies to the hospitals. Donor education of the populace especially targeting the youths is key to inculcating the right concept and culture of VNR blood donation. There is a great need for mass mobilization and retention of VNRD through effective evidence-based educational, cultural religious, and gender-based peculiarity intervention programmes and incentives. Hospital blood banks should have dedicated units saddled with this primary responsibility. Government and hospital managers should gear efforts toward the provision of equipment for component preparation and making it affordable. Also, TTI screening must step up to better and more sensitive screening techniques considering the large volume of blood required by patients and prevalence of TTIs.

## Figures and Tables

**Table 1:** Age and sex distribution of persons who donated blood during the study period.

Age groups	Male *n* (%)	Female *n* (%)	Total *n* (%)
18–25 years	3,905 (31.83)	148 (40.66)	4,053 (31.90)
26–35 years	4,729 (38.47)	156 (42.86)	4,885 (38.61)
36–45 years	3,041 (24.79)	48 (13.19)	3,089 (24.60)
46–55 years	552 (4.50)	10 (2.75)	562 (4.54)
56–65 years	38 (0.31)	1 (0.27)	39 (0.31)
>65 years	3 (0.024)	1 (0.27)	4 (0.03)
Total	12,268 (97.11)	364 (2.94)	12,632 (100.0)

**Table 2:** Age and sex distribution of donors across the categories of blood donation.

Age groups	Voluntary donors		Family replacement donors		Paid donors	
	Male (%)	Female (%)	Male (%)	Female (%)	Male (%)	Female (%)
18–25 years	84 (30.32)	16 (42.11)	2,580 (28.17)	122 (37.42)	1,591 (56.11)	0 (0.0)
26–35 years	113 (40.79)	17 (44.74)	3,634 (39.68)	143 (43.87)	1,036 (36.54)	0 (0.0)
36–45 years	69 (24.91)	4 (10.52)	2,483 (27.11)	46 (14.11)	196 (6.91)	0 (0.0)
46–55 years	10 (3.61)	1 (2.63)	429 (4.68)	13 (3.98)	12 (0.42)	0 (0.0)
56–65 years	0 (0.0)	0 (0.0)	28 (0.30)	1 (0.30)	0 (0.0)	0 (0.0)
>65 years	1 (0.3)	0 (0.0)	2 (0.02)	1 (0.30)	0 (0.0)	0 (0.0)
Total	277 (2.2)	38 (0.3)	9,156 (72.4)	326 (2.7)	2,835 (22.44)	0 (0.0)

**Table 3:** Distribution of blood group across persons who donated blood during the study period.

Blood group	Frequency	Percentage
A Rh−	38	0.3
A Rh+	1,360	12.1
B Rh−	39	0.3
B Rh+	1,057	9.4
AB Rh−	1	0.0
AB Rh+	56	0.5
O Rh−	403	3.6
O Rh+	8,239	73.6
Total	11,193	

**Table 4:** Seroprevalence of blood-borne diseases among persons screened during the study period.

TTI	Donor group	Positive (%)	Negative (%)	Total *N* (%)	Chi square	*p* value	Relative risk
	VNRD	1 (0.37)	268 (99.63)	269 (100.0)			1
HIV	FRD	87 (0.95)	9037 (99.05)	9124 (100.0)	4.952	0.084	2.56
	PAID	13 (0.49)	2625 (99.51)	2638 (100.0)			1.33
	Total	101 (0.82)	11930 (99.18)	12031 (100.0)			
	VNRD	4 (1.50)	263 (98.50)	267 (100.0)			1
HBV	FRD	230 (2.52)	8894 (97.48)	9124 (100.0)	14.940	0.001	1.68
	PAID	33 (1.25)	2602 (98.75)	2635 (100.0)			0.84
	Total	267 (2.22)	11759 (97.78)	12026 (100.0)			
	VNRD	8 (3.0)	259 (97.0)	267 (100.0)			1
HCV	FRD	199 (2.18)	8925 (97.82)	9124 (100.0)	9.9392	0.009	0.73
	PAID	33 (1.26)	2595 (98.74)	2628 (100.0)			0.42
	Total	240 (2.00)	11779 (98.0)	12019 (100.0)			
	VNRD	5 (1.87)	262 (98.13)	267 (100.0)			1
Syphilis	FRD	203 (2.22)	8921 (97.78)	9124 (100.0)	19.473	0.00006	1.19
	PAID	22 (0.84)	2598 (99.16)	2620 (100.0)			0.45
	Total	230 (1.91)	11781 (98.09)	12011			

**Table 5:** Site of blood donation/procurement.

Source of blood	Frequency	Percentage
Hospital blood bank (VNR and FR)	8,655	74.7
Paid	2,923	25.2
NBTS^*∗*^	3	0.03
Total	11,581	100.0

^*∗*^National Blood Transfusion Service.

**Table 6:** Pattern of usage of blood and blood components across clinical departments.

Blood component	O & G (%)	Paed (%)	Surgery (%)	A & E (%)	Medicine (%)	Haematology (%)
Whole blood	2,479 (99.67)	481 (94.31)	1,041 (98.49)	4,568 (99.96)	1,150 (94.10)	1,710 (98.55)
Red cell concentrate	1 (0.04)	7 (1.37)	4 (0.37)	1 (0.02)	32 (2.61)	3 (0.17)
Platelets	0 (0.00)	9 (1.76)	1 (0.09)	1 (0.02)	28 (2.29)	12 (0.69)
Fresh Plasma	7 (0.28)	13 (2.54)	11 (1.04)	0 (0.00)	12 (0.98)	10 (0.57)
Total	2,487 (21.47)	510 (4.40)	1,057 (9.12)	4,570 (39.46)	1,222 (10.55)	1,735 (14.98)

## Data Availability

The data set will be provided by the corresponding author on request.

## References

[1] World Health Organization and International Federation of Red Cross and Red Crescent Societies (2010). *Towards 100% Voluntary Blood Donation: A Global Framework for Action*.

[2] Nwogoh B., Awodu O. A. (2012). Blood donation in Nigeria: standard of the donated blood. *Lab Physicians*.

[3] WHO (2016). *Blood Safety and Availability*.

[4] Aneke J., Okocha C. (2017). Blood transfusion safety; current status and challenges in Nigeria. *Asian Journal of Transfusion Science*.

[5] Okoroiwu H. U., Okafor I. M., Asemota E. A., Okpokam D. C. (2018). Seroprevalence of transfusion-transmissible infections (HBV, HCV, syphilis, and HIV) among prospective blood donors in a tertiary health care facility in Calabar, Nigeria; an eleven years evaluation. *BMC Public Health*.

[6] Oluyinka O. O., Tong H. V., Bui Tien S. (2015). Occult hepatitis B virus infection in Nigerian blood donors and hepatitis B virus transmission risks. *PLoS One*.

[7] Olotu A. A., Oyelese A. O., Salawu L., Audu R. A., Okwuraiwe A. P., Aboderin A. O. (2016). Occult hepatitis B virus infection in previously screened, blood donors in ile-Ife, Nigeria: implications for blood transfusion and stem cell transplantation. *Virology Journal*.

[8] Bala J. A., Kawo A. H., Mukhtar M. D., Ai S., Magaji N., Aliyu I. A. (2012). Sani MN 1 prevalence of hepatitis C virus infection among blood donors in some selected hospitals in Kano, Nigeria. *International Research Journal of Microbiology*.

[9] Aleruchi O., Peterside N. F., Ezekoye C. C. (2014). Seroprevalence of HIV infection among blood donors at the university of port harcourt teaching hospital, rivers state, Nigeria. *Global journal of biology, Agriculture and Health Sciences*.

[10] https://www.premiumtimesng.com/news/headlines/467648-blood-donor-day-severe-consequences-as-Nigeria-gets-27-of-annual-blood-need.html.

[11] Okocha C., Ogbenna A., Ezeama N., Aneke J., Ezeh T. (2019). Pattern of blood procurement and utilization in a university hospital in Southeast Nigeria. *Annals of Tropical Pathology*.

[12] Enosolease M. E., Imarengiaye C. O., Awodu O. A. (2004). Donor blood procurement and utilisation at the university of Benin teaching hospital, Benin city. *African Journal of Reproductive Health*.

[13] Shittu A. O., Olawumi H. O., Omokanye K. O., Ogunfemi M. K., Adewuyi J. O. (2019). The true status of family replacement donors in a tertiary hospital blood service in central Nigeria. *Africa Sanguine*.

[14] Olaiya M. A., Alakija W., Ajala A., Olatunji R. O. (2004). Knowledge, attitudes, beliefs and motivations towards blood donations among blood donors in Lagos, Nigeria. *Transfusion Medicine*.

[15] Agbovi K.-K., Kolou M., Fétéké L., Haudrechy D., North M.-L., Ségbéna A.-Y. (2006). Étude des connaissances, attitudes et pratiques en matière de don de sang. Enquête sociologique dans la population de Lomé (Togo). *Transfusion Clinique et Biologique*.

[16] https://www.who.int/news-room/fact-sheets/detail/blood-safety-and-availability.2021.

[17] Erhabor O. l., Isaac Z. l., Abdulrahaman Y. (2013). Female gender participation in the blood donation process in resource poor settings: case study of sokoto in north western Nigeria. *Journal of Blood Disorders & Transfusion*.

[18] Madrona D. P., Herrera M. D. F., Jiménez D. P., Giraldo S. G., Campos R. R. (2014). Women as whole blood donors: offers, donations and deferrals in the province of Huelva, south-western Spain. *Blood Transfus*.

[19] Misje A. H., Bosnes V., Heier H. E. (2010). Gender differences in presentation rates, deferrals and return behaviour among norwegian blood donors. *Vox Sanguinis*.

[20] Kouao M. D., Dembelé B., N’Goran L. K. (2012). Reasons for blood donation deferral in sub-sahara Africa: experience in ivory coast. *Transfusion*.

[21] Ekwere T. A., Ino-Ekanem M., Motilewa O. O., Ibanga I. A. (2014). Pattern of blood donor deferral in a tertiary hospital, South-south, Nigeria: a three-year study review. *International Journal of Blood Transfusion and Immunohematology*.

[22] Nwogoh B., Isoa E., Ikpomwen O. (2011). Donor blood procurement and the risk of transfusion transmissible viral infections in a tertiary health facility in South-South Nigeria. *Nigerian Medical Journal*.

[23] Siraj N., Achila O. O., Issac J. (2018). Seroprevalence of transfusion-transmissible infections among blood donors at national blood transfusion service, Eritrea: a seven-year retrospective study. *BMC Infectious Diseases*.

[24] Anifowoshe A. T., Owolodun O. A., Akinseye K. M., Iyiola O. A., Oyeyemi B. F. (2017). Gene frequencies of ABO and Rh blood groups in Nigeria: a review. *Egyptian Journal of Medical Human Genetics*.

[25] Hamed C. T., Bollahi M. A., Abdelhamid I. (2011). Frequencies and ethnic distribution of ABO and Rh (D) blood groups in Mauritania: results of first nationwide study. *International Journal of Immunogenetics*.

[26] Garg P., Upadhyay S., Chufal S. S., Hasan Y., Tayal I. (2014). Prevalance of ABO and rhesus blood groups in blood donors: a study from a tertiary care teaching hospital of kumaon region of uttarakhand. *Journal of Clinical and Diagnostic Research: Journal of Clinical and Diagnostic Research*.

[27] Khattak I. D., Khan T. M., Khan P., Shah S. M., Khattak S. T., Ali A. (2008). Frequency of ABO and rhesus blood group in district Swat, Pakistan. *Journal of Ayub Medical College*.

[28] Erhabor O., Erhabor T., Adias T. C., Ikechukwu Polycarp I. (2019). Distribution of clinically relevant blood group antigens among Nigerians and the management of rhesus D negative pregnancies: implications for haemolytic disease of the foetus and newborn and haemolytic transfusion reactions. *Human Blood Group Systems and Haemoglobinopathies*.

